# Phylogeography and Genetic Variation of *Triatoma dimidiata*, the Main Chagas Disease Vector in Central America, and Its Position within the Genus *Triatoma*

**DOI:** 10.1371/journal.pntd.0000233

**Published:** 2008-05-07

**Authors:** María Dolores Bargues, Debora R. Klisiowicz, Fernando Gonzalez-Candelas, Janine M. Ramsey, Carlota Monroy, Carlos Ponce, Paz María Salazar-Schettino, Francisco Panzera, Fernando Abad-Franch, Octavio E. Sousa, Christopher J. Schofield, Jean Pierre Dujardin, Felipe Guhl, Santiago Mas-Coma

**Affiliations:** 1 Departamento de Parasitología, Facultad de Farmacia, Universidad de Valencia, Burjassot, Valencia, Spain; 2 Departamento de Genética, Instituto Cavanilles de Biodiversidad y Biología Evolutiva, Universidad de Valencia, Valencia, Spain; 3 Centro Regional de Investigación en Salud Pública (CRISP), Instituto Nacional de Salud Pública (INSP), Tapachula, Chiapas, México; 4 Universidad San Carlos, Laboratorio de Entomología Aplicada y Parasitología, Guatemala; 5 Laboratorio Central de Referencia para Enfermedad de Chagas y Leishmaniasis, Secretaría de Salud, Tegucigalpa, Honduras; 6 Laboratorio Biología de Parásitos, Departamento de Microbiología y Parasitología, Facultad de Medicina, U.N.A.M., México D.F., México; 7 Centro de Investigaciones sobre Enfermedades Infecciosas, Instituto Nacional de Salud Pública, Cuernavaca, Morelos, México; 8 Sección Genética Evolutiva, Facultad de Ciencias, Universidad de la República, Montevideo, Uruguay; 9 Biodiversity Laboratory–Medical Entomology, Centro de Pesquisa Leônidas & Maria Deane, Fiocruz, Manaus, Brazil; 10 Center for Research and Diagnosis of Parasitic Diseases, Faculty of Medicine, University of Panama, Panama City, Republic of Panama; 11 Department of Infectious and Tropical Diseases, London School of Hygiene and Tropical Medicine, London, United Kingdom; 12 Institut de Recherche pour le Developpement (IRD), Representative Office, French Embassy, Bangkok, Thailand; 13 Centro de Investigaciones en Microbiología y Parasitología Tropical (CIMPAT), Facultad de Ciencias, Universidad de los Andes, Bogotá, Colombia; Universidad de Buenos Aires, Argentina

## Abstract

**Background:**

Among Chagas disease triatomine vectors, the largest genus, *Triatoma*, includes species of high public health interest. *Triatoma dimidiata*, the main vector throughout Central America and up to Ecuador, presents extensive phenotypic, genotypic, and behavioral diversity in sylvatic, peridomestic and domestic habitats, and non-domiciliated populations acting as reinfestation sources. DNA sequence analyses, phylogenetic reconstruction methods, and genetic variation approaches are combined to investigate the haplotype profiling, genetic polymorphism, phylogeography, and evolutionary trends of *T. dimidiata* and its closest relatives within *Triatoma*. This is the largest interpopulational analysis performed on a triatomine species so far.

**Methodology and Findings:**

Triatomines from Mexico, Guatemala, Honduras, Nicaragua, Panama, Cuba, Colombia, Ecuador, and Brazil were used. *Triatoma dimidiata* populations follow different evolutionary divergences in which geographical isolation appears to have had an important influence. A southern Mexican–northern Guatemalan ancestral form gave rise to two main clades. One clade remained confined to the Yucatan peninsula and northern parts of Chiapas State, Guatemala, and Honduras, with extant descendants deserving specific status. Within the second clade, extant subspecies diversity was shaped by adaptive radiation derived from Guatemalan ancestral populations. Central American populations correspond to subspecies *T. d. dimidiata*. A southern spread into Panama and Colombia gave the *T. d. capitata* forms, and a northwestern spread rising from Guatemala into Mexico gave the *T. d. maculipennis* forms. *Triatoma hegneri* appears as a subspecific insular form.

**Conclusions:**

The comparison with very numerous *Triatoma* species allows us to reach highly supported conclusions not only about *T. dimidiata*, but also on different, important *Triatoma* species groupings and their evolution. The very large intraspecific genetic variability found in *T. dimidiata sensu lato* has never been detected in a triatomine species before. The distinction between the five different taxa furnishes a new frame for future analyses of the different vector transmission capacities and epidemiological characteristics of Chagas disease. Results indicate that *T. dimidiata* will offer problems for control, although dwelling insecticide spraying might be successful against introduced populations in Ecuador.

## Author Summary

Chagas disease is a serious parasitic disease of Latin America. Human contamination in poor rural or periurban areas is mainly attributed to haematophagous triatomine insects. *Triatoma* includes important vector species, as *T. dimidiata* in Central and Meso-America. DNA sequences, phylogenetic methods and genetic variation analyses are combined in a large interpopulational approach to investigate *T. dimidiata* and its closest relatives within *Triatoma*. The phylogeography of *Triatoma* indicates two colonization lineages northward and southward of the Panama isthmus during ancient periods, with *T. dimidiata* presenting a large genetic variability related to evolutionary divergences from a Mexican-Guatemalan origin. One clade remained confined to Yucatan, Chiapas, Guatemala and Honduras, with extant descendants deserving species status: *T*. sp. aff. *dimidiata*. The second clade gave rise to four subspecies: *T. d. dimidiata* in Guatemala and Mexico (Chiapas) up to Honduras, Nicaragua, Providencia island, and introduced into Ecuador; *T. d. capitata* in Panama and Colombia; *T. d. maculipennis* in Mexico and Guatemala; and *T. d. hegneri* in Cozumel island. This taxa distinction may facilitate the understanding of the diversity of vectors formerly included under *T. dimidiata*, their different transmission capacities and the disease epidemiology. *Triatoma dimidiata* will offer more problems for control than *T. infestans* in Uruguay, Chile and Brazil, although populations in Ecuador are appropriate targets for insecticide-spraying.

## Introduction

American trypanosomiasis or Chagas disease is widespread in Latin America from Mexico to Chile and southern Argentina. Although present estimates of 10 to 12 million people infected with the haemoflagellate protozoan species *Trypanosoma cruzi* represent 6–8 million fewer cases than those reported in the 1980s [1], it remains one of the most serious parasitic diseases of the Americas for its social and economic impact [2]. Although it can also be transmitted by blood transfusion or across the placenta from infected mothers, most human contamination is attributed to insect vectors in poor rural or periurban areas of Central and South America [1].

Chagas disease vectors are haematophagous reduviid (Hemiptera: Heteroptera) insects belonging to the subfamily Triatominae. Species of Triatominae are usually grouped into 17 genera forming five tribes, although other arrangements have been proposed. Of these, Alberproseniini, Bolboderini, Cavernicolini and Rhodniini are considered monophyletic, whereas Triatomini is considered polyphyletic [3]. Among the latter, most of the species (over 70) are included in the genus *Triatoma*, among which two main clades appear in ribosomal DNA (rDNA) sequence phylogenies, corresponding to species of North and Central America and species of South America separated prior to the closing of the isthmus of Panama about 3 million years ago [4]–[6]. Moreover, *Triatoma* species are distributed in three main groupings: the Rubrofasciata group (mainly North American and Old World species), the Phyllosoma group (mainly Mesoamerican and Caribbean), and the Infestans group (mainly South American), each including different complexes and subcomplexes in a classification which is progressively updated according to new genetic and morphometric data [7].

A priori, all of the over 130 species currently recognized within Triatominae seem capable of transmitting *T. cruzi*. Among the species of greatest epidemiological significance as domestic vectors, three belong to the genus *Triatoma*: *T. infestans* and *T. brasiliensis* from South America, and *T. dimidiata*, distributed in Meso- and Central America from Mexico down to Colombia, Venezuela, Ecuador and northern Peru [3].

*Triatoma dimidiata* can be found in sylvatic, peridomestic and domestic habitats. Non-domiciliated populations may act as reinfestation sources and become involved in the transmission of the parasite to humans [8],[9]. This species includes morphologically variable populations [10],[11]. A molecular comparison of Triatominae, including many Central American species of the Phyllosoma complex by means of rDNA second internal transcribed spacer (ITS-2) sequences demonstrated an unusual intraspecific sequence variability in a few *T. dimidiata* populations studied. This study even revealed differences consistent with a specific status for populations from the Yucatan peninsula, Mexico [4]–[6], thus opening a debate. A large number of recent, multidisciplinary studies using RAPD-PCR, genital structures, morphometrics of head characters, and antennal phenotypes have shown that variation within this species seems much greater than previously considered [8], [12]–[16]. Morphometric and cuticular hydrocarbon analyses suggest that a sylvatic population from Lanquin, Guatemala, is undergoing a speciation process [13],[17]. Chromosomal variation and genome size suggest that *T. dimidiata* may represent a complex of cryptic species (i.e. morphologically indistinguishable, yet reproductively isolated taxa) [18].

The aim of the present work is to analyze the intraspecific variability, haplotype profiling, phylogeography and genetic polymorphism of populations of the species *T. dimidiata*, to get a new framework able to facilitate the future understanding of the diferring peculiarities of this crucial vector species throughout its broad geographical distribution. This may also help in understanding the related differences in characteristics of Chagas disease transmission and epidemiology, as well as in responses to control initiatives in the countries concerned. After a deep analysis, it was considered that the most convenient approach would be obtained by using an appropriate marker able to furnish significant information about evolutionary trends of variation on which to construct the new baseline. This new baseline should be, whenever possible, of sufficient weight as to allow its conclusions to be reflected at systematic-taxonomic level.

For this purpose, the rDNA was preferred over mitochondrial DNA (mtDNA) because of its mendelian inheritance, evolutionary rates and overall recognized usefulness in systematics in all metazoan organism groups because of including sequences which allow to distinguish between species and between subspecies units. The better fitting of rDNA for molecular systematics has already been emphasized in large reviews on rDNA/mtDNA marker comparisons in insects [19]. Ribosomal DNA includes excellent genetic markers, because (i) the rDNA operon is tandemly repeated and present in sufficiently high quantities among the genome of an individual thus facilitating sequencing procedures; (ii) the different genes and spacers of the rDNA follow a concerted evolution which, with sufficient time, effectively homologizes the many copies of nuclear rDNA within a genome [20]; this gives rise to a uniformity of their sequences within all individuals of a population and becomes extremely useful from an applied point of view, because it is sufficient to obtain the sequence of only one individual to characterize the local population it belongs to, that is, all other individuals of that population will present the same sequence; (iii) the usefulness of rDNA genes and spacers as genetic markers at different evolutionary levels have already been verified on a large number of very different eukaryotic organism groups including insects, and consequently extensive knowledge on the different rDNA fragments is available [21]. rDNA sequence comparisons offer valuable information about the evolutionary events in triatomine lineages and, by deducing the routes of spreading of triatomine populations, they may also shed light on the ability of different species to colonize new areas [5].

Within rDNA, ITS-2 was selected as marker because of its well-known usefulness at species and subspecies levels, including the differentiation of taxa within problematic groups, as is the case of those comprising cryptic or sibling species of other insect groups [22]–[24]. Moreover, the sequences of the ITS-2 have already proved to be a useful tool in the analysis of species, subspecies, hybrids and populations, and for inferring phylogenetic relationships in Triatominae in general [4],[5],[6],[25],[26].

In order to be able to assess the ITS-2 evolutionary processes followed by *T. dimidiata* populations, the ITS-2 sequences of many members of the Phyllosoma, Rubrofasciata and Infestans groups were obtained and analyzed. For this purpose, a large number of rDNA ITS-2 sequences of *Triatoma* species from numerous geographic origins in Mexico, Guatemala, Honduras, Nicaragua, Panama, Cuba, Colombia, Ecuador, and Brazil was studied. Thus, the nucleotide divergence limits between taxa within the lineage of the genus *Triatoma* could be established. The present study on *T. dimidiata* is the largest interpopulational analysis performed on a triatomine species so far.

## Materials and Methods

### Triatomine materials

**Figure 1:**
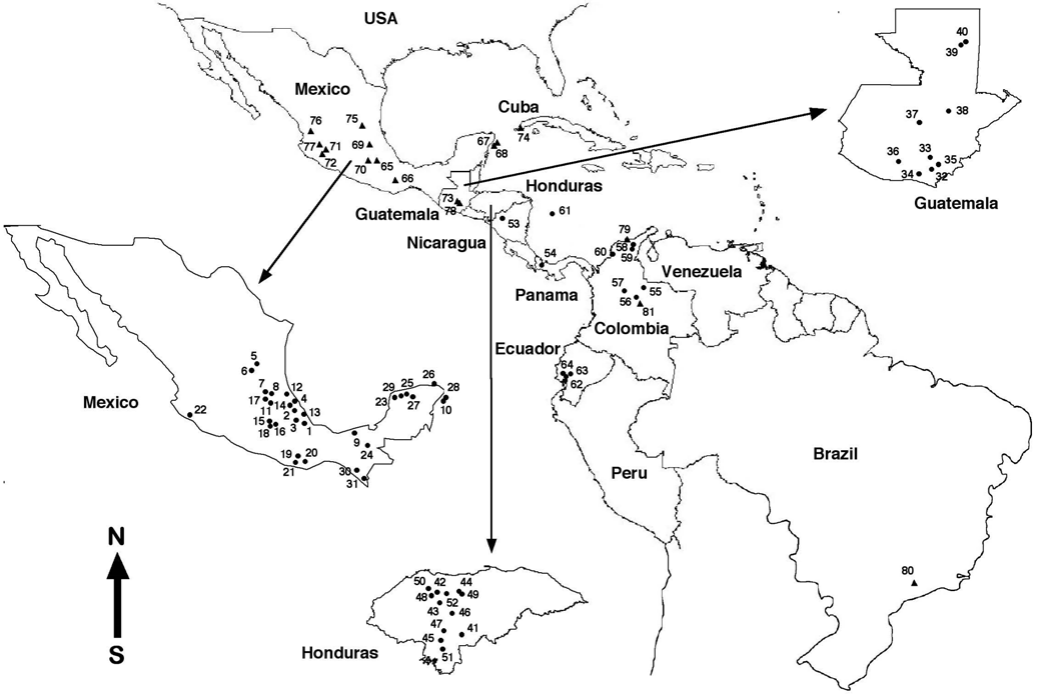
Geographical distribution of the sampling sites furnishing the triatomine materials. Numbers correspond to sampling sites listed in Table 1. • = *Triatoma dimidiata*; ▴ = other *Triatoma* species studied.

A total of 165 triatomine specimens representing 13 *Triatoma* species of the Phyllosoma, Rubrofasciata and Infestans groups, among which 137 specimens representing *T. dimidiata* from 64 different geographic origins, were used for sequencing, genetic variation and phylogenetic analyses (Table 1; Figure 1). The systematic classification recently proposed for the genus *Triatoma* [7] is used here throughout.

**Table 1:** *Triatoma* species and samples studied, including ITS-2 sequence length and AT composition (in percentage).

Country	Map No.	Preliminary classification	Sampling sites	Haplotype code	Sequence length	% AT
**PHYLLOSOMA GROUP: PHYLLOSOMA COMPLEX**						
**1) ** ***TRIATOMA DIMIDIATA*** **: 31 different haplotype sequences/137 specimens studied:**						
**MEXICO**	1	*T. dimidiata*	Atoyac Tlacorrancho, Veracruz	T.dim-H18	496	75.81
n = 41	2	*T. dimidiata*	Atoyac-Manzanillo, Veracruz	T.dim-H18	496	75.81
	3	*T. dimidiata*	Atoyac-Cordoba, Veracruz	T.dim-H18	496	75.81
	4	*T. dimidiata*	Ursulo-Galan, Veracruz	T.dim-H18	496	75.81
	5	*T. dimidiata*	Tanchahuil, San Luis Potosí	T.dim-H18	496	75.81
	6	*T. dimidiata*	Barrio Tzitzi, San Luis Potosí	T.dim-H18	496	75.81
	7	*T. dimidiata*	Huejutla, Hidalgo (3)	T.dim-H18	496	75.81
	8	*T. dimidiata*	Atlapexco, Hidalgo	T.dim-H18	496	75.81
	9	*T. dimidiata*	El Rosario, Tabasco	T.dim-H18	496	75.81
	10	*T. dimidiata*	Cozumel island, Quintana Roo	T.dim-H18	496	75.81
	11	*T. dimidiata*	Acomul, Hidalgo	T.dim-H18	496	75.81
	12	*T. dimidiata*	Mesa de Tlanchinol, Veracruz	T.dim-H19	494	75.71
	13	*T. dimidiata*	La Luz, Veracruz	T.dim-H19	494	75.71
	14	*T. dimidiata*	Emiliano Zapata, Veracruz	T.dim-H20	495	75.76
	15	*T. dimidiata*	Morelos	T.dim-H21	497	75.85
	16	*T. dimidiata*	Cajones, Morelos	T.dim-H21	497	75.85
	17	*T. dimidiata*	Huehuetla, Hidalgo	T.dim-H22	494	75.71
	18	*T. dimidiata*	Chalcatzingo, Morelos	T.dim-H23	496	75.60
	19	*T. dimidiata*	Santiago Cuixtla, Oaxaca	T.dim-H23	496	75.60
	20	*T. dimidiata*	Hierba Santa, Oaxaca	T.dim-H23	496	75.60
	21	*T. dimidiata*	Nopala, Oaxaca	T.dim-H23	496	75.60
	22	*T. dimidiata*	Alcaraces, Cuauhtemoc, Colima	T.dim-H24	496	75.40
	23	*T. dimidiata*	Paraíso, Yucatán (3)	T.dim-H25	493	75.66
	24	*T. dimidiata*	Palenque, Chiapas	T.dim-H25	493	75.66
	23	*T. dimidiata*	Paraíso, Yucatán	T.dim-H26	489	75.46
	23	*T. dimidiata*	Paraíso, Yucatán	T.dim-H27	494	75.51
	25	*T. dimidiata*	Yaxkukul,Yucatán	T.dim-H28	493	75.66
	26	*T. dimidiata*	Holbox island, Quintana Roo	T.dim-H28	493	75.66
	23	*T. dimidiata*	Paraíso, Yucatán	T.dim-H28	493	75.66
	27	*T. dimidiata*	Izamal, Yucatán	T.dim-H28	493	75.66
	28	*T. dimidiata*	Cozumel island, Quintana Roo (3)	T.dim-H28	493	75.66
	23	*T. dimidiata*	Paraíso, Yucatán	T.dim-H28	493	75.66
	29	*T. dimidiata*	Chablekal, Mérida, Yucatán	T.dim-H31	489	75.25
	30	*T. dimidiata*	Mapastepec, Chiapas	T.dim-H1	497	76.06
	31	*T. dimidiata*	Tapachula, Chiapas	T.dim-H3	497	76.26
**GUATEMALA**	32	*T. dimidiata*	Jutiapa, Jutiapa (4)	T.dim-H1	497	76.06
n = 37	33	*T. dimidiata*	Agua Zarca, Jutiapa	T.dim-H1	497	76.06
	34	*T. dimidiata*	Pueblo Nuevo Viñas, Santa Rosa	T.dim-H1	497	76.06
	35	*T. dimidiata*	Piedra Pintada, Jutiapa (3)	T.dim-H1	497	76.06
	33	*T. dimidiata*	Agua Zarca, Jutiapa	T.dim-H2	496	76.01
	36	*T. dimidiata*	Escuintla, Escuintla (3)	T.dim-H2	496	76.01
	37	*T. dimidiata*	San Andrés Sajcabaja, Quiché	T.dim-H2	496	76.01
	34	*T. dimidiata*	Pueblo Nuevo Viñas, Santa Rosa	T.dim-H2	496	76.01
	33	*T. dimidiata*	Agua Zarca, Jutiapa (2)	T.dim-H3	497	76.26
	36	*T. dimidiata*	Escuintla, Escuintla	T.dim-H3	497	76.26
	34	*T. dimidiata*	Pueblo Nuevo Viñas, Santa Rosa	T.dim-H3	497	76.26
	37	*T. dimidiata*	San Andrés Sajcabaja, Quiché	T.dim-H4	497	76.85
	34	*T. dimidiata*	Pueblo Nuevo Viñas, Santa Rosa	T.dim-H8	497	76.06
	35	*T. dimidiata*	Aldea Piedra Pintada, Jutiapa	T.dim-H8	497	76.06
	38	*T. dimidiata*	Lanquín, Alta Verapaz (4)	T.dim-H10	496	76.01
	39	*T. dimidiata*	Chultún, Yaxhá, Petén (2)	T.dim-H18	496	75.81
	37	*T. dimidiata*	San Andrés Sajcabaja, Quiché (2)	T.dim-H18	496	75.81
	40	*T. dimidiata*	Yaxhá, Petén	T.dim-H25	493	75.66
	40	*T. dimidiata*	Yaxhá, Petén (2)	T.dim-H28	493	75.66
	40	*T. dimidiata*	Yaxhá, Petén (3)	T.dim-H28	493	75.66
	40	*T. dimidiata*	Yaxhá, Petén	T.dim-H30	491	75.56
**HONDURAS**	41	*T. dimidiata*	Güinope, El Paraiso	T.dim-H1	497	76.06
n = 20	42	*T. dimidiata*	El Tablon, Yoro (2)	T.dim-H2	496	76.01
	43	*T. dimidiata*	El Zapote, Yoro	T.dim-H2	496	76.01
	44	*T. dimidiata*	El Salitre, Yoro	T.dim-H2	496	76.01
	45	*T. dimidiata*	El Cacao, Francisco Morazán (2)	T.dim-H2	496	76.01
	46	*T. dimidiata*	Orica, Francisco Morazán	T.dim-H2	496	76.01
	47	*T. dimidiata*	Tegucigalpa, Francisco Morozán (2)	T.dim-H2	496	76.01
	48	*T. dimidiata*	Corral Falso, Yoro (2)	T.dim-H2	496	76.01
	49	*T. dimidiata*	El Salitre, Montaña, Yoro	T.dim-H2	496	76.01
	50	*T. dimidiata*	Subirama, Yoro	T.dim-H2	496	76.01
	51	*T. dimidiata*	San José, Choluteca	T.dim-H6	496	76.01
	48	*T. dimidiata*	Corral Falso, Yoro	T.dim-H9	496	75.81
	43	*T. dimidiata*	El Zapote, Yoro	T.dim-H9	496	75.81
	50	*T. dimidiata*	Subirama, Yoro	T.dim-H9	496	75.81
	50	*T. dimidiata*	Subirama, Yoro	T.dim-H29	494	75.71
	52	*T. dimidiata*	El Paraiso, Yoro	T.dim-H29	494	75.71
**NICARAGUA**	53	*T. dimidiata*	Madriz	T.dim-H7	497	75.65
n = 1						
**PANAMA**	54	*T. dimidiata*	Boquete, Chiriqui (3)	T.dim-H16	497	76.06
n = 4	54	*T. dimidiata*	Boquete, Chiriqui	T.dim-H17	495	75.96
**COLOMBIA**	55	*T. dimidiata*	Pore, Casanare	T.dim-H11	497	75.85
n = 31	56	*T. dimidiata*	Boavita, Boyacá (13)	T.dim-H11	497	75.85
	57	*T. dimidiata*	San Joaquín, Santander (3)	T.dim-H11	497	75.85
	58	*T. dimidiata*	Com. Los Kuises, SNSM Magdalena	T.dim-H12	495	75.76
	56	*T. dimidiata*	Boavita, Boyacá (4)	T.dim-H12	495	75.76
	59	*T. dimidiata*	Sierra Nevada, Santa Marta (4)	T.dim-H12	495	75.76
	56	*T. dimidiata*	Boavita, Boyacá	T.dim-H13	493	75.66
	60	*T. dimidiata*	San Onofre, Sucre (insectary) (2)	T.dim-H14	497	76.06
	56	*T. dimidiata*	Boavita, Boyacá	T.dim-H15	497	75.65
	61	*T. dimidiata*	Providencia island	T.dim-H1	497	76.06
**ECUADOR**	62	*T. dimidiata*	Guayas, Guayaquil	T.dim-H5	497	75.85
n = 3	63	*T. dimidiata*	Cerro del Carmen, Guayas, Guayaquil	T.dim-H5	497	75.85
	64	*T. dimidiata*	Pedro Carbo, Guayaquil	T.dim-H6	496	76.01
**2) ** ***TRIATOMA BASSOLSAE*** **: 1 sequence/2 specimens studied:**						
**MEXICO**	65	*T. bassolsae*	Acatlán, Puebla (2)	T.bas-H1	490	76.94
n = 2						
**3) ** ***TRIATOMA BOLIVARI*** **: 1 sequence/1 specimen studied:**						
**MEXICO**	66	*T. bolivari*	Oaxaca, Oaxaca	T.bol-H1	501	76.85
**4) ** ***TRIATOMA HEGNERI*** **: 2 sequences/5 specimens studied:**						
**MEXICO**	67	*T. hegneri*	Ruinas S.Gervasio, Cozumel isl., Q. Roo	T.heg-H1	496	75.81
n = 5	68	*T. hegneri*	Cedral, Cozumel isl., Quintana Roo (3)	T.heg-H1	496	75.81
	68	*T. hegneri*	Cedral, Cozumel isl., Quintana Roo	T.heg-H2	496	76.01
**5) ** ***TRIATOMA MEXICANA*** **: 1 sequence/1 specimen studied:**						
**MEXICO**	69	*T.mexicana*	Itatlaxco, Hidalgo	T.mex-H1	492	75.61
**6) ** ***TRIATOMA PALLIDIPENNIS*** **: 1 sequence/3 specimens studied:**						
**MEXICO**	70	*T. pallidipennis*	Chalcatzingo, Morelos	T.pal-H1	491	76.98
n = 3	71	*T. pallidipennis*	San Gabriel, Jalisco	T.pal-H2	490	76.94
	72	*T. pallidipennis*	Tecalitlan, Jalisco	T.pal-H2	490	76.94
**7) ** ***TRIATOMA RYCKMANI*** **: 1 sequence/2 specimens studied:**						
**GUATEMALA**	73	*T. ryckmani*	El Progreso, El Progreso (2)	T.ryc-H1	500	76.00
n = 2						
**PHYLLOSOMA GROUP: FLAVIDA COMPLEX**						
**8) ** ***TRIATOMA FLAVIDA*** **: 1 sequence/4 specimens studied:**						
**CUBA**	74	*T. flavida*	Peninsula of Guanahacabibes (4)	T.fla-H1	493	78.70
n = 4						
**RUBROFASCIATA GROUP: RUBROFASCIATA COMPLEX: LECTICULARIA SUBCOMPLEX**						
**9) ** ***TRIATOMA GERSTAECKERI*** **: 1 sequence/1 specimen studied:**						
**MEXICO**	75	*T. gerstaeckeri*	Tanchahuil, S. Luis Potosí	T.ger-H1	483	76.81
**10) ** ***TRIATOMA RUBIDA*** **: 1 sequence/2 specimens studied:**						
**MEXICO**	76	*T. rubida*	Mocorito, Nayarit	T.rub-H1	516	77.71
n = 2	77	*T. rubida*	San Martin, Jalisco	T.rub-H1	516	77.71
**RUBROFASCIATA GROUP: PROTRACTA COMPLEX**						
**11) ** ***TRIATOMA NITIDA*** **: 1 sequence/1 specimen studied:**						
**GUATEMALA**	78	*T. nitida*	El Progreso, El Progreso	T.nit-H1	490	76.33
**INFESTANS GROUP: INFESTANS COMPLEX: MACULATA SUBCOMPLEX**						
**12) ** ***TRIATOMA MACULATA*** **: 1 sequence/4 specimens studied:**						
**COLOMBIA**	79	*T. maculata*	Santa Marta, Magdalena (4)	T.mac-H1	488	78.28
n = 4						
**INFESTANS GROUP: INFESTANS COMPLEX: RUBROVARIA SUBCOMPLEX**						
**13) ** ***TRIATOMA ARTHURNEIVAI*** **: 1 sequence/2 specimens studied:**						
**BRAZIL**	80	*T.arthurneivai*	Espirito Santo do Pinhal	T.art-H1	486	77.98
n = 2			Sao Paulo (Fiocruz) (2)			

### Sequencing of rDNA ITS-2

For DNA extraction, one or two legs fixed in ethanol 70% from each specimen were used and processed individually, as previously described [5],[27]. Total DNA was isolated by standard techniques [28] and stored at −20°C until use. The complete ITS-2 fragment was PCR amplified using 4–6 µl of genomic DNA for each 50 µl reaction. Amplifications were generated in a Peltier thermal cycler (MJ Research, Watertown, MA, USA), by 30 cycles of 30 sec at 94°C, 30 sec at 50°C and 1 min at 72°C, preceded by 30 sec at 94°C and followed by 7 min at 72°C. PCR products were purified with Ultra Clean™ PCR Clean-up DNA Purification System (MoBio, Solana Beach, CA, USA) according to the manufacturer's protocol and resuspended in 50 µl of 10 mM TE buffer (10 mM Tris-HCl, 1 mM EDTA, pH 7.6). Sequencing was performed on both strands by the dideoxy chain-termination method, and with the Taq dye-terminator chemistry kit for ABI 3730 and ABI 3700 capillary system (Perkin Elmer, Foster City, CA, USA), using the same amplification PCR primers [6].

### Triatomine haplotype code nomenclature

The haplotype (H) terminology used in the present paper follows the nomenclature for composite haplotyping (CH) recently proposed [25]. Accordingly, ITS-2 haplotypes (H) are noted by numbers (Table 1).

### Sequence alignment

Sequences were aligned using CLUSTAL-W version 1.83 [29] and MEGA 3.1 [30], and assembly was made with the Staden Package [31]. The alignment was carried out using the Central, Meso and South American *Triatoma* species studied together with other species and populations whose sequences are available in GenBank: *T. phyllosoma* (Accession Number AJ286881), *T. pallidipennis* (AJ286882), *T. longipennis* (AJ286883), *T. picturata* (AJ286884), and *T. mazzotti* (AJ286885) (Phyllosoma group, Phyllosoma complex); *T. barberi* (AJ293590) (Rubrofasciata group, Protracta complex) [5],[6]; *T. rubrovaria* H1 (AJ557258) [32], *T. infestans* CH1A (AJ576051), and *T. sordida* (AJ576063) [25]. The ITS-2 sequence of *Rhodnius prolixus* (Triatominae: Rhodniini) (AJ286882) [6] was used as outgroup.

### Data deposition footnote

The GenBank (http://www.ncbi.nlm.nih.gov/Genbank) accession numbers for the new ITS-2 rDNA sequences discussed in this paper are: 31 haplotypes of *T. dimidiata* (AM286693–AM286723), *T. bassolssae* AM286724, *T. bolivari* (AM286725), 2 haplotypes of *T. hegneri* (AM286726, AM286727), *T. mexicana* (AM286728), 2 haplotypes of *T. pallidipennis* (AM286729, AM286730), *T. ryckmani* (AM286731), *T. flavida* (AM286732), *T. gerstaeckeri* (AM286734), *T. rubida* (AM286735), *T. nitida* (AM286733), *T. maculata* (AJ582027), and *T. arthurneivai* (AM286736).

### Phylogenetic inference

Phylogenies were inferred by maximum-likelihood (ML) using PAUP*4.0b10 [33] and PHYMLv2.4.4 [34]. Maximum-likelihood parameters and the evolutionary model were determined using the hierarchical Likelihood Ratio Test (hLRTs) and the Akaike Information Criterion (AIC) [35],[36] implemented in Modeltest 3.7 [37] in conjunction with PAUP*4b10. To assess the reliability of the nodes in the ML tree, a bootstrap analysis using 1000 pseudo-replicates was made with PHYML. Since haplotype sequences for *T. dimidiata* individuals (populations) are quite similar and potentially subject to homoplasy and recombination, alternative procedures to phylogenetic tree reconstruction revealing their relationships were tested. Therefore, a median-joining network analysis [38] was performed using Network version 4.1.1.2 (available from Fluxus Technology Ltd., http://www.fluxus-engineering.com) with the variable positions in the multiple alignment of the different ITS-2 haplotypes from *T. dimidiata* populations.

Alternative methods of phylogenetic reconstruction allowing an evaluation of the support for each node were also applied. A distance-based phylogeny using the neighbor-joining (NJ) algorithm [39] with the ML pairwise distances was obtained. Statistical support for the nodes was evaluated with 1000 bootstrap replicates, with and without removal of gapped positions. Finally, a Bayesian phylogeny reconstruction procedure was applied to obtain posterior probabilities (BPP) for the nodes in the ML tree. We used the same evolutionary model as above implemented in MrBayes 3.1 [40] with four chains during 1,000,000 generations and trees were sampled every 100 generations. The last 9,000 trees were used to obtain the consensus tree and posterior probabilities.

### Genetic variation studies

Genetic variation within and among populations of *T. dimidiata* was evaluated using DnaSP version 4 [41] and Arlequin 2000 [42]. Summary parameters include those based on the frequency of variants (haplotype number and diversity) as well as some taking genetic differences among variants into account (gene diversity, polymorphic sites). A hierarchical analysis of molecular variance (AMOVA) was performed using Arlequin. This analysis provides estimates of variance components and F-statistics [43] analogs reflecting the correlation of haplotype diversity at different levels of hierarchical subdivision. Unlike other approaches for partitioning genetic variation based on the analysis of variance of gene frequencies, AMOVA takes into account the genetic relatedness between molecular haplotypes. The hierarchical subdivision was made at three levels. At the top level, different groups were defined on the basis of the phylogenetic relationships for the different *T. dimidiata* haplotypes obtained. The second level corresponded to countries of sampling within each of these groups, and the third level corresponded to the different haplotypes found in each country within group. AMOVA reports components of variance at the three levels under consideration (among groups, among countries within groups, and within countries within groups) as well as F-statistics analogs. Under the present scheme, F_ST_ is viewed as the correlation of random haplotypes within countries within groups, relative to that of random pairs of haplotypes drawn from the whole species, F_CT_ as the correlation of random haplotypes within groups, relative to that of random pairs of haplotypes drawn from the whole species, and F_SC_ as the correlation of the molecular diversity of random haplotypes within countries within groups, relative to that of random pairs of haplotypes drawn from the corresponding group [44]. Although in the program used (only currently available for molecular variance analysis) the choice for establishing an intermediate level is fully arbitrary and has no influence on the final result of the comparison between units at the higher level, these same analyses were repeated by considering each haplotype, which may encompass several individuals, as a separate group for this intermediate level, because it could be argued that geopolitical country borders was not an appropriate choice despite its interest from the point of view of the control of Chagas disease. The statistical significance of fixation indices was tested using a non-parametric permutation approach [44]. Genetic differentiation between pairs of populations was evaluated by means of F-statistics [43]. Exact tests of population differentiation were performed [45]. Slatkin's linearized F_ST_'s [46],[47] procedure was also followed to obtain estimates of pairwise equilibrium migration rates, both among groups, among countries within groups, and within countries for those cases in which haplotypes from more than one group were present.

## Results

### Sequence Analyses of *Triatoma dimidiata* Populations

**Figure 2:**
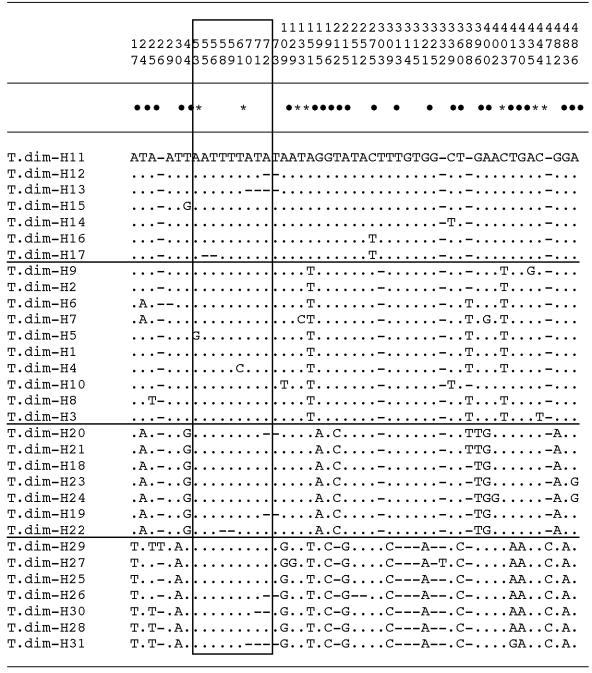
Interhaplotype sequence differences found in the rDNA ITS-2 of the *Triatoma dimidiata* populations analyzed. Numbers (to be read in vertical) refer to positions obtained in the alignments made with CLUSTAL-W 1.8 and MEGA 3.3. . = identical; * = singelton sites (7); • = parsimony informative positions (24); − = insertion/deletion. Rectangled area = microsatellite region. Horizontal lines separate the four major *T. dimidiata* haplotype groupings according to sequence analyses.

The 137 ITS-2 sequences revealed the existence of 31 different haplotypes in the *T. dimidiata* studied (T.dim-H1 to T.dim-H31) (see Tables 1 and 2 for localities and countries). Their length was 489–497 base pairs (bp) (mean, 495.10) with a relative AT-biased nucleotide composition of 75.25–76.85% (75.72%). Sequence similarity analysis of these 31 haplotypes revealed four distinct groupings: grouping 1 (T.dim-H1 to T.dim-H10); grouping 2 (T.dim-H11 to T.dim-H17); grouping 3 (T.dim-H18 to T. dim-H24); and grouping 4 (T. dim-H25 to T. dim-H31) (Figure 2). These four groupings appear linked to concrete wide geographical areas including neighboring countries and regions. The only exception is Providencia Island, which, although part of Colombia, is located 720 km off the northern coast of Colombia but only 240 km off the western coast of Nicaragua. No haplotype presents a very broad geographical distribution.

**Table 2:** Distribution of *Triatoma dimidiata* ITS-2 haplotypes (H) per country and locality.

Country	Locality	Sample	H	H	H	H	H	H	H	H	H	H	H	H	H	H	H	H	H	H	H	H	H	H	H	H	H	H	H	H	H	H	H
		size	1	2	3	4	5	6	7	8	9	10	11	12	13	14	15	16	17	18	19	20	21	22	23	24	25	26	27	28	29	30	31
**COLOMBIA**	**Dpto. Casanare**	1											1																				
**(n = 31)**	**Dpto. Boyaca**	19											13	4	1		1																
	**Dpto. Santander**	3											3																				
	**Santa Marta**	5												5																			
	**Sucre**	2														2																	
	**Providencia Isl.**	1	1																														
**PANAMA (n = 4)**	**Chiriqui**	4																3	1														
**MEXICO**	**Veracruz**	7																		4	2	1											
**(n = 41)**	**Oaxaca**	4																					1		3								
	**Morelos**	2																					1		1								
	**San Luis Potosi**	1																		2													
	**Hidalgo**	6																		5				1									
	**Tabasco**	1																		1													
	**Colima**	1																								1							
	**Chiapas**	3	1		1																						1						
	**Yucatan**	10																									3	1	1	4			1
	**Cozumel Isl.**	4																		1										3			
	**Holbox Isl.**	1																												1			
**HONDURAS**	**Yoro Yoro**	13		8							3																				2		
**(n = 20)**	**El Porvenir**	2		2																													
	**Orica**	1		1																													
	**Tegucigalpa**	2		2																													
	**Ginope**	1	1																														
	**San Jose**	1						1																									
**ECUADOR (n = 3)**	**Guayaquil**	3					2	1																									
**NICARAGUA (n = 1)**	**Madriz**	1							1																								
**GUATEMALA**	**Agua Sarca**	4	1	1	2																												
**(n = 37)**	**Esquintla**	4		3	1																												
	**Quiche**	4		1		1														2													
	**Pueblo Nuevo**	4	1	1	1					1																							
	**Peten**	9																		2							1			5		1	
	**Jutiapa**	8	7							1																							
	**Lanquin**	4										4																					
	**TOTAL**	137	12	19	5	1	2	2	1	2	3	4	17	9	1	2	1	3	1	17	2	1	2	1	4	1	5	1	1	13	2	1	1

Sequence groupings: grouping 1 (T.dim-H1 to T.dim-H10); grouping 2 (T.dim-H11 to T.dim-H17); grouping 3 (T.dim-H18 to T.dim-H24); and grouping 4 (T.dim-H25 to T.dim-H31).

The alignment of the 31 *T. dimidiata* haplotype sequences was 501 bp-long, of which 450 characters were constant and 24 were parsimony-informative. The interrupted microsatellite (AT)_4–5_ TTT (AT)_5–7_ was detected between positions 47 and 73 in all specimens studied. Variability in this microsatellite region and their respective sequence positions are noted in Figure 2.

The 51 nucleotide variable positions detected including gaps represented a 10.18% of polymorphic sites. The seven haplotypes T.dim-H25 to T.dim-H31 are responsible for this high genetic divergence (Figure 2). This genetic divergence decreases considerably when two separate alignments are performed: (i) the first includes T.dim-H1 to T.dim-H24 from all the seven countries shows a divergence of 5.62% in a 498-bp-long alignment, including 28 nucleotide variable positions, of which 6 (1.20%) were transitions (ti), 13 (2.61%) transversions (tv) and 9 (1.81%) insertions/deletions (indels); (ii) the second includes T.dim-H25 to T.dim-H31 from only three countries (Mexico: localities of Yucatan, Chiapas, Cozumel Island and Holbox Island; Guatemala: Peten; Honduras: Yoro Yoro) shows a divergence of 2.42% in a 495-bp-long alignment, with 12 nucleotide variable positions, of which 2 ti (0.40%) and 10 are indels (2.02%).

### Sequence Analyses in the Phyllosoma and Rubrofasciata Groups

ITS-2 sequences of *T. bassolsae*, *T. bolivari*, *T. hegneri*, *T. mexicana*, *T. pallidipennis*, *T. ryckmani*, *T. flavida*, *T. nitida*, *T. gerstaeckeri*, and *T. rubida*, including haplotype length and AT content are listed in Table 1. The comparison analyses which include these ITS-2 sequences and those of the Phyllosoma and Rubrofasciata groups (available in GenBank) provided 48 different haplotypes. Their alignment resulted in a total of 551 characters including gaps, of which 365 sites were constant and 99 parsimony-informative.

All the *T. dimidiata* haplotypes clearly differed from the Phyllosoma, Flavida, Protacta and Rubrofasciata complex species included in this analysis. *Triatoma bassolsae* differed in only one deletion in position 489 from *T. pallidipennis* of Morelos, Mexico (AJ286882). The *T. pallidipennis* sequence obtained represents a new haplotype (T.pal-H2) differing in only one deletion in position 31 from *T. picturata* and *T. longipennis*. The haplotype alignment of *T. bassolsae*, *T. longipennis*, *T. mazzotti, T. picturata*, *T. pallidipennis* and *T. phyllosoma* was 490 bp long showing a relatively small genetic diversity of 1.83%, with only 5 mutations (1.02%) and 4 indels (0.81%). The two *T. hegneri* haplotypes differ between each other in only 1 ti and, when compared with *T. dimidiata* H18 to H24 from Mexico and Guatemala, nucleotide differences found were only 1 ti and 2 tv.

### Sequence Analyses in the Infestans Group

ITS-2 sequences of *T. maculata* and *T arthurneivai*, including haplotype length and AT content are listed in Table 1.

The ITS-2 of *T. maculata* fits very well within sequences of the Infestans complex species studied in the present work, a total of 6–19 (13.7) mutations, namely 6–11 (7.25) ti and 0–10 (6.5) tv, appearing when comparing the five Infestans complex species in question. The material of *Triatoma arthurneivai* here analyzed is very close to *T. rubrovaria* H1 (AJ557258), showing only 6 nucleotide differences (1.22%), of which only 1 ti and 5 indels.

### Phylogenetic Analyses

Two different phylogenetic approaches were performed with the 31 *T. dimidiata* haplotypes, both yielding coincident results. A maximum likelihood tree was reconstructed using the best model of evolution as determined by the lowest AIC, which was GTR+I (−Ln = 887.089), being the proportion of invariable sites (I) of 0.166. Three groups appeared with high support values indicating that their differentiation was not due to random sampling of a low variable sequence (tree not shown). The large group 1 encompassed haplotypes from all the countries, whereas groups 2 (Mexico and Guatemala) and 3 (Mexico, Guatemala and Honduras) were more geographically restricted.

**Figure 3:**
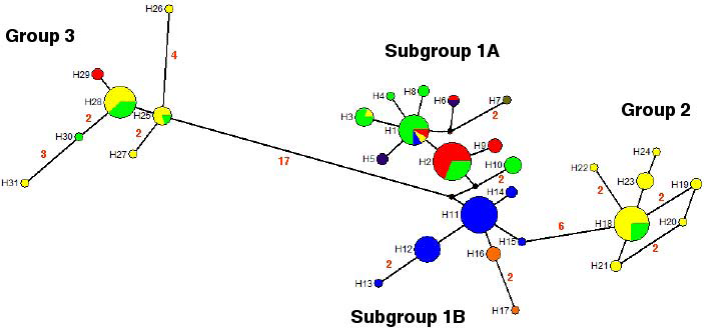
Median network for *Triatoma dimidiata* haplotypes based on rDNA ITS-2 sequences. The area of each haplotype is proportional to the total sample. Small black-filled circles represent haplotypes not present in the sample. Mutational steps between haplotypes are represented by a line. More than one mutational step is represented by numbers. H = haplotype. Blue: Colombia; orange: Panama; yellow: Mexico; red: Honduras; lilac: Ecuador; ocher: Nicaragua; green: Guatemala.

Alternatively, a median-joining network was reconstructed with the 31 different *T. dimidiata* sequences using the variable sites in the multiple alignment (Figure 3). This network showed the same three groups found in the ML tree. Group 1 occupies a central position in the network and is the most widespread and variable group, so that it most likely corresponds to the ancestral or source set. This is further reinforced by the direct relationship between this group and the two others, more geographically restricted and encompassing fewer variants, group 2 including samples from Mexico and Guatemala, and group 3 including samples from these two countries and Honduras. The group 1 source set would in turn be derived from group 3, which might be interpreted as a geographically restricted relict according to the phylogeographic results. Moreover, sequence variants in group 1 are clustered in two different subgroups, with genetic and geographical borders: subgroup 1A includes sequences from Colombian Providencia island, Ecuador, Guatemala, Honduras, Mexico (only South of Chiapas) and Nicaragua; subgroup 1B encompasses sequences from continental Colombia and Panama. The two closest sequences of each subgroup differ in two sites, which might correspond to haplotypes not found in this sampling.

**Figure 4:**
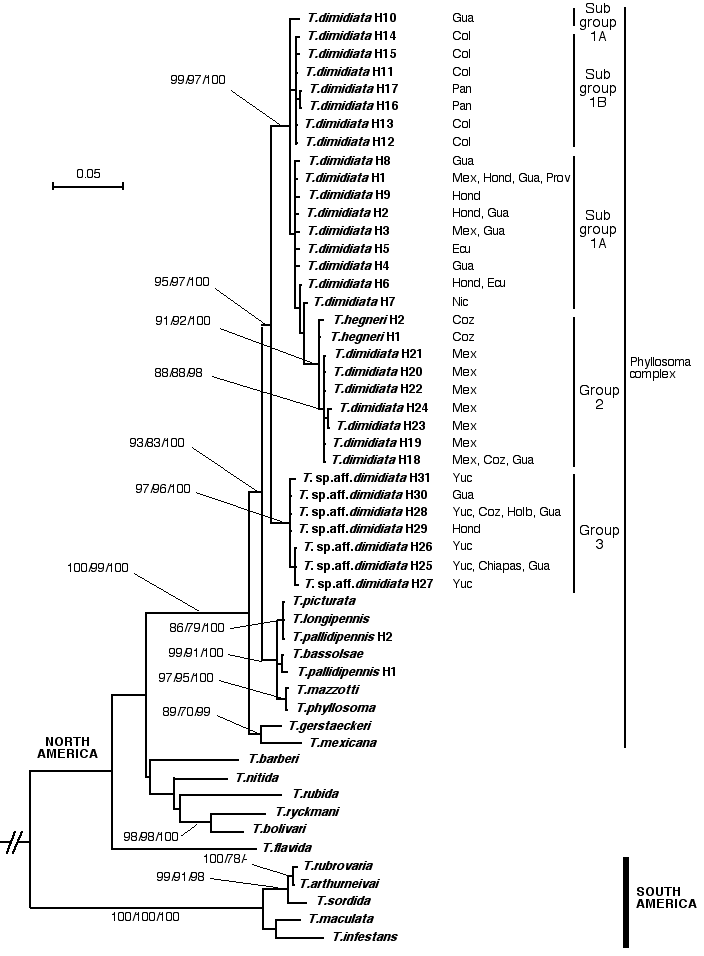
Phylogenetic ML tree of *Triatoma* species and haplotypes within the Phyllosoma, Rubrofasciata and Infestans groups. The scale bar indicates the number of substitutions per sequence position. Support for nodes a/b/c: a: bootstrap with ML reconstruction using PhyML with 1000 replicates; values larger than 70%; b: bootstrap with NJ reconstruction using PAUP with ML distance and 1000 replicates; values larger than 70%; c: Bayesian posterior probability with ML model using MrBayes; values larger than 90%.

The relevance of the ITS-2 differences among these *T. dimidiata* groups and subgroups was assessed by comparison with other *Triatoma* species. Therefore, a multiple, 562-nucleotide-long alignment was obtained by incorporating 22 additional ITS-2 sequences. This set includes 53 ITS-2 sequences of *Triatoma* species and, using *R. prolixus* as outgroup, a ML tree was obtained (−Ln = 2648.5129) using the HKY+G model, according to the AIC results with a gamma distribution shape parameter = 0.58. This tree (Figure 4) shows that:

- the 31 *T. dimidiata* haplotypes appear within a highly supported clade (95/97/100 in ML/NJ/BPP), distributed as follows: a first large subclade, also very well supported (99/97/100), comprising subgroup 1A, subgroup 1B, group 2, and group 3 of the network analysis; subgroup 1A (sequence grouping 1 = T.dim-H1 to T.dim-H10) includes populations from Central America (Honduras, Nicaragua, Guatemala and scattered haplotypes from Mexico, Ecuador and Providence Island); interestingly, the haplotype T.dim-H10 corresponding to phenetically peculiar specimens found in cave-dwellings of Lanquin, Guatemala, appears independent although related to the rest with very high supports; subgroup 1B (sequence grouping 2 = T.dim-H11 to T.dim-H17) comprises populations from continental Colombia and Panama and appears as a monophyletic haplotype cluster; group 2 (sequence grouping 3 = T.dim-H18 to T.dim-H24) shows a well supported branch (91/92/100) and comprises populations from Mexico (Gulf coast, high plains, and Cozumel island) and Guatemala, including the two *T. hegneri* haplotypes; the second large clade is also highly supported (97/96/100), corresponding to group 3 (sequence grouping 4 = T.dim-H25 to T.dim-H31) and includes populations from the Yucatan peninsula, Holbox and Cozumel islands and northern Chiapas (Mexico), northern Honduras and northern Guatemala;
- *T. bassolsae* clusters together with *T. phyllosoma*, *T. mazzotti*, *T. longipennis*, *T. picturata* and *T. pallidipennis* with very high support (99/91/100 in ML/NJ/BPP) in a sister clade of *T. dimidiata*; the separated location of the two *T. pallidipennis* haplotypes indicates the marked similarity of all these taxa;
- *T. mexicana* and *T. gerstaeckeri* cluster together in a group basal to both *T. dimidiata* and *T. phyllosoma* clades; the extremely high values (100/99/100) supporting the monophyletic clade including *T. mexicana*, *T. gerstaeckeri*, *T. phyllosoma* and close species, and *T. dimidiata*, are worth emphasizing;
- *T. barberi*, *T. nitida*, *T. rubida*, *T. ryckmani* and *T. bolivari* cluster in an unresolved branch, within which only *T. ryckmani* and *T. bolivari* appear related with a high support; the insular species *T. flavida* from Cuba appears as a basal lineage although with insufficient support values;
- finally, the South American species *T. rubrovaria*, *T. arthurneivai*, *T. sordida*, *T. maculata* and *T. infestans* cluster together with the highest support.

*Triatoma dimidiata* groupings appeared well supported, with very high bootstrap proportions (BP>90%) using ML and neighbor-joining reconstruction and the highest Bayesian posterior probabilities (BPP = 100%). Similar levels were found for other well established *Triatoma* species, many of which showed substantially lower support values in the three statistical measurements employed. However, other species presented no ITS-2 nucleotide differences (*T. picturata* and *T. longipennis*; *T. mazzotti* and *T. phyllosoma*).

### Genetic Variation Analyses

The phylogenetic analyses showed that samples from the same country may belong to different clusters. This result, on its own, is not enough to demonstrate the biological distinctiveness of the corresponding populations. Sampled individuals may represent a minor fraction of the total genetic variability in a highly heterogeneous population and the sampling procedure might have resulted, by pure chance, in the observed clustering of some variants. Given that each of these clusters holds some genetic variability of its own, the first task was to evaluate whether the observed groupings were significantly different from each other, in terms of genetic variation, by partitioning the observed genetic variability at three different levels: among groups, among populations (countries) within groups, and within populations. A hierarchical analysis of molecular variance was used to test the null hypothesis of no genetic differentiation among groups considering variation at lower levels. This procedure was first applied to *T. dimidiata* sequences using three levels as defined above (Table 3a). Most of the genetic variation found was allocated to the among groups level (80.24% of the total variation), with much lower portions of variation assigned to differences among populations within groups level (11.71%) and within populations level (8.05%), although both were still statistically significant after 1000 pseudo-random samples generated for testing. This indicates that, despite genetic variation within and among populations at these three levels, there is a substantial amount of genetic differentiation among them that justifies their consideration as separate groupings for further analysis. The same results were obtained, notwithstanding small numerical differences due to the different numbers of groups, when haplotypes instead of countries were considered at the intermediate level (Table S1). The geographical fitting represents in fact no surprise at all, taking into account that the distribution of *T. dimidiata* covers different countries which are more or less aligned following a north-south axis because of the relatively slenderness of the Central American bridge. Hence, as any of the two versions of the analyses conveys the same information and leads to the same conclusions, and which one should be reported is simply a matter of opinion, the first considering countries becomes practically more useful because Chagas disease control measures are organized at national level.

**Table 3:** Summary of analysis of molecular variance for *Triatoma dimidiata* populations.

Source of variation	d.f.	Sum of squares	Variance components	Percentage of variation	Fixation Indices
a)					
Among groups	2	528.273	6.732 Va	80.24	F_CT_ = 0.802***
Among populations within groups	10	86.820	0.982 Vb	11.71	F_ST_ = 0.920***
Within populations	123	83.047	0.675 Vc	8.05	F_SC_ = 0.593***
Total	135	698.140	8.389		
b)					
Among groups	1	68.257	1.4785	60.15	F_CT_ = 0.602*
Among populations within groups	6	15.547	0.3007	12.23	F_ST_ = 0.724***
Within populations	77	52.267	0.6788	27.62	F_SC_ = 0.307***
Total	84	136.071	2.4580		
c)					
Among groups	3	596.530	5.890	86.84	F_CT_ = 0.868***
Among populations within groups	9	18.563	0.218	3.21	F_ST_ = 0.900***
Within populations	123	83.047	0.675	9.95	F_SC_ = 0.244***
Total	135	698.140	6.783		

(a) Three groups (1, 2, and 3), (b) two subgroups (1A vs 1B), and (c) four groups/subgroups (1A, 1B, 2 and 3) were considered as indicated in the text. Populations within groups correspond to countries of sampling. ^***^: P<0.001; ^**^: P<0.01. d.f. = degrees of freedom.

The median-joining network reconstructed with the 31 different *T. dimidiata* ITS-2 sequences revealed the existence of three distinct groups (groups 1, 2 and 3), the first of which further subdivided into two subgroups 1A and 1B. The same AMOVA procedure was applied to ascertain whether these two subgroups could be considered as distinct populations or not. The results (Table 3b) indicate that a significant fraction (60.15%) of the total genetic variation corresponds to differences between these two subgroups which, correspondingly, could be considered as separate populations for the ensuing analyses.

Based on the four groups/subgroups previously described in the median-joining network, a summary of relevant population genetic parameters for *T. dimidiata* is presented in Table 4. Genetic variation in *T. dimidiata* populations was quite evenly distributed, with similar levels of nucleotide and haplotype diversities in the four groups/subgroups considered. Nevertheless, for all the parameters studied, subgroup 1A presented higher values than the rest, although significance of the differences was only obtained for haplotype diversity. A similar summary is shown for each country sample within groups in Table S2.

**Table 4:** Summary of population genetic variation parameters from ITS-2 haplotypes in the *Triatoma dimidiata* populations.

Parameter	Group1	Subgroup1A	Subgroup1B	Group2	Group3
Gene copies	85	51	34	27	24
Haplotypes	17	10	7	7	7
Polymorphic sites	23	13	9	7	11
Hap. diversity	0.8782	0.797	0.686	0.6353	0.6775
Std. error	0.0178	0.040	0.065	0.0972	0.0902
Pairwise diff. mean	3.2398	1.707	1.524	1.1510	1.6377
Std. error	1.6872	1.014	0.938	0.7670	1.0007
Nucleot diversity	0.0065	0.003	0.003	0.0023	0.0033
Std. error	0.0037	0.002	0.002	0.0017	0.0023
θ (Het)	6.0371	3.105	1.668	1.3162	1.5990
S.D. θ (Het)	1.1075	0.822	0.523	0.5710	0.6892
θ (k)	6.1156	3.444	2.385	2.7281	2.9510
95 % C.I. for θ (k)	3.476,10.432	1.668,6.785	1.009,5.308	1.134,6.223	1.213,6.838
θ (S)	3.1911	2.445	1.223	0.5189	0.8034
S.D. θ (S)	1.1040	0.976	0.636	0.3844	0.5094
θ (π)	3.2398	1.707	1.524	1.1510	1.6377
S.D. θ (π)	1.8694	1.125	1.043	0.8553	1.1155
Tajima's D	−1.261ns	−1.572*	−1.553*	−0.536ns	−0.6435ns
Ewens-Watterson	0.132ns	0.219ns	0.334ns	0.388ns	0.3507ns
Fu's Fs	−3.401ns	−2.601ns	−1.111ns	−2.426*	−1.4665ns

θ = effective mutation rate estimated from equilibrium heterozygosity [θ(Het)], number of alleles [θ(k)], number of polymorphic sites [θ(S)] and nucleotide diversity [θ(π)]. The last 3 rows correspond to different statistics of neutrality at the population level. S.D. = standard deviation; C.I. = confidence interval. NS: P>0.05; ^*^ = P<0.05.

Different estimates of θ were obtained based on the expected heterozygosity, the expected number of alleles, the number of polymorphic sites and the nucleotide diversity. The four estimates were quite consistent for the four groups/subgroups and they agreed in assigning a larger value to subgroup 1A.

Differences in the genetic composition of the four groups/subgroups 1A, 1B, 2 and 3 have previously been shown to be statistically significant according to analyses of molecular variance. A further evaluation of this distinctiveness was made (Table 3c), in which the four groups/subgroups were considered for the AMOVA, in correspondence with the previous results. In this case, the amount of among-group variation rose to 86.84% of the total variation, whereas among population within groups and within population levels they were substantially lower, 3.21% and 9.95% respectively.

Genetic differences within and among the ITS-2 locus for *T. dimidiata* samples were further explored through pairwise comparisons, and estimates of average pairwise differences within and among the four groups/subgroups considered were obtained (Table 5). Subgroup 1A presented the largest value for within-group pairwise differences. The within-population values were much lower than among-populations comparisons. Among the latter, the smallest number of differences was found between subgroup 1A and 1B, in correspondence with their close phylogenetic relationship. Subgroup 1B was the one with the lowest overall number of pairwise differences, slightly below 1A. On the contrary, the highest value of pairwise differentiation corresponds to group 3, with almost 20 differences (corrected estimate) when compared with any other group.

**Table 5:** Population average pairwise differences in *Triatoma dimidiata* populations.

	Group 1	Subgroup1A	Subgroup1B	Group2	Group3
Group 1	3.240	-	-	9.953	20.719
Subgroup1A	-	1.707	4.922	10.325	21.118
Subgroup1B	-	3.307	1.524	9.397	20.120
Group2	7.758	8.896	8.059	1.151	26.875
Group3	18.280	19.446	18.539	25.481	1.638

Above diagonal: Average number of pairwise differences between populations (π_XY_). Diagonal elements: average number of pairwise differences within population (π_X_). Below diagonal: corrected average pairwise difference (π_XY_−(π_X_+π_Y_)/2).

Within groups genetic differentiation was evaluated by computation of pairwise F_ST_ values for populations defined by country of origin (Table S3). Since all groups/subgroups, with the only exception of subgroup 1A, are characterized by one large (n>10) and several small (n<10) populations, significance values for test of genetic differentiation have to be interpreted cautiously. Hence, there is no apparent differentiation between two populations in subgroup 1B (Colombia2, n = 30, and Panama, n = 4) and similarly in group 2 (Mexico2, n = 23, and Guatemala2, n = 4). The only significant value found in group 3 corresponds to Honduras3 (n = 2) and Guatemala3 (n = 7), for which F_ST_ = 0.529, P<0.05. None of these two populations presented significant differentiation with respect to the largest population in this group, Mexico3 (n = 15). Subgroup 1A includes two large populations, Honduras1 (n = 18) and Guatemala1 (n = 26), which presented a highly significant F_ST_ = 0.193, P<0.001. Although this value, under the assumption of migration-drift equilibrium, corresponds to an estimate of 2.1 migrants per generation between both populations, which would be enough to prevent their complete differentiation, such estimations shall be verified by using larger samples and markers better suited for population genetics analyses. Comparisons between each of these two populations and the smaller ones in subgroup 1A revealed that Honduras1 differed from Mexico1, Guatemala1 was different from Ecuador and Nicaragua, and none of them differed from the only two individuals from Providencia island. Similar comparisons for all pairs of populations assigned to different groups/subgroups resulted in highly significant F_ST_ values (Table S4).

## Discussion

### *Triatoma dimidiata, T.* sp. aff. *dimidiata* and *T. hegneri*

The highest intraspecific ITS-2 variability (absolute nucleotide differences including indels) known in Triatomini members is 2.70% (13/482) in *T. infestans* specimens collected throughout the very wide geographical distribution of this species [25]. Hence, the result of 10.18% ( = 51/501) detected in *T. dimidiata* (Figure 2) appears to be pronouncedly outside the limits of the intraspecific variability range known for *Triatoma* species. Group 3 is the main responsible for such differences (Table 5) and shows a high 2.42% divergence within itself, suggesting an old origin in the light of the relatively reduced geographical area of distribution of these haplotypes in Mexico (Yucatan, Chiapas, Cozumel Island and Holbox Island), Guatemala (Peten) and Honduras (Yoro) only. The time of divergence between group 3 and other *T. dimidiata* populations was estimated to be of 5.9–10.5 million years ago (Mya) according to a molecular clock analysis based on rDNA evolutionary rates [4].

The divergence of 5.62% shown by the other 24 ITS-2 haplotypes (Figure 2) also appears to be too large, in spite of the wide geographical area they occupy from Mexico down to Ecuador, suggesting a speciation process. However, population average pairwise differences between subgroup 1A, subgroup 1B and group 2 are markedly lower than between these three and group 3 (Table 5), and intragroup differences do fall within the above-mentioned Triatomini range: 2.61% within subgroup 1A, 2.41% within subgroup 1B, and 2.01% within group 2.

**Figure 5:**
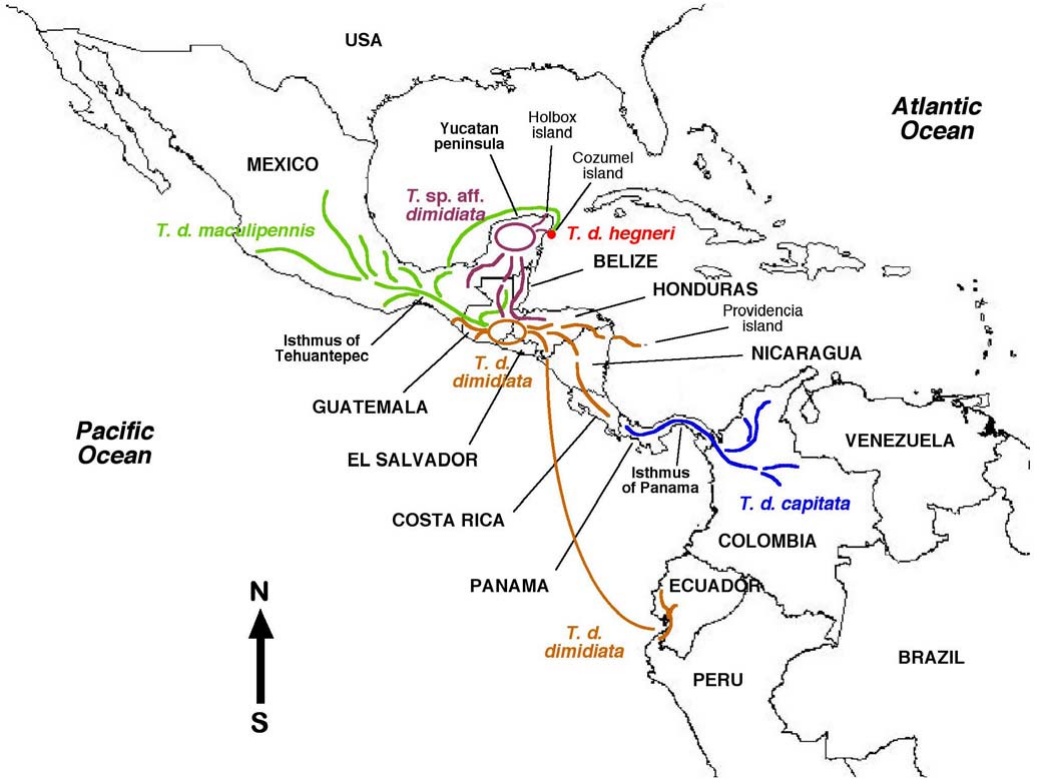
Phylogeography of *Triatoma dimidiata sensu lato.* Distribution and spreading routes of *T. d. dimidiata*, *T. d. capitata*, *T. d. maculipennis*, *T. d. hegneri* and *Triatoma* sp. aff. *dimidiata* in Mesoamerica, Central America and the northwestern part of South America are represented according to network analyses and genetic variation studies based on rDNA ITS-2 sequences.

Results indicate that several *T. dimidiata* populations are following different evolutionary divergences in which geographical isolation appears to have had an important influence (Figure 5). A phenotypic consequence of that process had been observed by other specialists before, who wrote about an assemblage of morphologically variable populations [10]. More recently, significant head shape differences between populations showed a separation between northern, intermediate and southern collections of *T. dimidiata* and also support an evolutionary divergence of populations within this species [13].

Three subspecies were distinguished on the basis of morphological differences [48],[49]: (i) *T. d. dimidiata* concerns the first description of the species in Peru (no type specimen available; no type locality assigned, but undoubtedly from northern Peru, probably around the locality of Tumbes, near Ecuador) and corresponds to most of the Central American forms; (ii) *T. d. maculipennis* was proposed for specimens from Mexico (type specimen in Zoologisches Museum Berlin) and corresponds to forms with relatively short heads and large eyes; and (iii) *T. d. capitata* was proposed for large size specimens typified by longer heads and smaller eyes originally found in Colombia (type specimen in the Academy of Sciences of California). However, these subspecies became later synonymized after results of a morphological re-examination which were interpreted as evidence of a clinal variation along a north-south axis [50],[51].

Present ITS-2 sequences and corresponding phylogenetic and genetic variation analyses support the appropriateness to (i) differentiate group 3 as a species of its own (here simply designed as *T*. sp. aff. *dimidiata* to avoid further systematic confusion with *T. dimidiata*, according to taxonomic rules), and (ii) re-assign subspecific status for subgroup 1A, subgroup 1B and group 2. Results of the present study do not support the rise of the above-mentioned subspecific taxa to species level for the time being, although it is evident that in the three cases relatively long divergence processes have taken place. Similar genetic studies with other molecular markers may contribute to a more complete assessment of these evolutionary isolation and speciation processes.

The taxon *T*. sp. aff. *dimidiata* concerns group 3. This species seems to represent a relatively relict species with a distribution restricted to the Mexican flat areas of the Yucatan peninsula and the northern part of Chiapas state, the northern lowland of Guatemala (and probably also Belize), and only one altitude-adapted haplotype (T.dim-H29) in its most extreme border populations in northern Honduras. The most widely spread haplotype T.dim-H28 is also present in the small island of Holbox and the large island of Cozumel, both near the Yucatan coast, suggesting that this haplotype should be considered the oldest of this species. This species is also of public health importance because of its capacity to transmit Chagas disease [52],[53] and the control problems it poses [54],[55].

The taxon *T. d. dimidiata* corresponds to subgroup 1A and populations mainly from Guatemala and Honduras and secondarily Mexico, Nicaragua and Ecuador. The population of the Colombian island of Providence undoubtedly derives from the most widely dispersed haplotype T.dim-H1 on the nearest Caribbean coastal area of Central America and not from continental Colombia. The present populations in Ecuador may derive from introduced specimens originally from the Guatemala-Honduras-Nicaragua region, relatively recently introduced by humans [4], very probably in the period of the early colonialization of northwestern South America by the Spanish ‘conquistadores’ in which exchange activities between Central American settlements and the Peruvian Tumbes area took place [56]. The type specimens of the original description of the species in northern Peru might also belong to populations derived from such man-made introductions from Central America. The haplotype T.dim-H10 of Lanquin, Alta Verapaz, Guatemala appears in the network analysis as directly derived from an ancestor which gave rise to the subspecies *T. d. dimidiata*. An isolation phenomenon in caves may explain the albinic characteristics of the specimens presenting this haplotype. These cavernicole specimens from Alta Verapaz have already shown their peculiarity in morphometric and cuticular hydrocarbon studies [13],[17].

The taxon *T. d. capitata* corresponds to subgroup 1B and populations from Colombia and Panama. The isthmus of Panama and the separation/joining process of South and North America towards the end of the Pliocene (3–5 Mya) [57], in a period in which several more or less closely separated islands appeared and evolved up to their fusion into the isthmus, should have played a major role in the isolation and subsequent divergence of these southernmost *T. dimidiata* populations. The lack of relationship between the haplotypes of Ecuador and those of Colombia is worth mentioning, as the geographical closeness of these two countries could have given rise to the erroneous hypothesis of Colombian forms having derived from Ecuadorian populations. In a recent study of three populations of sylvatic, peridomestic and domestic *T. dimidiata* from Colombia, the estimated low genetic distances based on RAPD analyses did not discriminate the populations studied, indicating that they maintain the genetic identity of a single recent common ancestor [9].

The taxon *T. d. maculipennis* corresponds to group 2 and populations mainly from Mexico, but rarely found in Guatemala. According to the network analysis, this subspecies seems to have derived from group 1 probably by isolation in the Mexican part northward from the isthmus of Tehuantepec. Similarly as for other organisms including insects [58], the mountainous Sierra Madre chain throughout southern Mexico and Guatemala areas near the Pacific coast probably played also a role in that isolation process through an area where *T*. sp. aff. *dimidiata* did not represent a competition barrier, as *T*. sp. aff. *dimidiata* appears to be preferentially a low altitude species in these two countries.

Southern Mexico (including the Yucatan peninsula and Chiapas state) and almost the whole country of Guatemala (at least ten departments) constitute a crucial evolutionary area, where a high number of taxa, including *T. d. dimidiata*, *T. d. maculipennis*, and *T*. sp. aff. *dimidiata*, overlap. In a morphometric analysis, populations from San Luis Potosi and Veracruz in Mexico were indistinguishable while clearly different from populations from Yucatan in Mexico and Peten in Guatemala [14]. The former correspond to *T. d. maculipennis* and the latter to *T*. sp. aff. *dimidiata*. In Guatemala, a high degree of genetic variation in *T. dimidiata sensu lato* was shown by RAPD-PCR [12], demonstrating a limited gene flow between different provinces, although barriers between the Atlantic and Pacific drainage slopes did not appear to be significant limiters of a gene flow, according to a hierarchical analysis.

Chromosome analyses and DNA genome size revealed the existence of three different cytotypes with different geographical distributions [18]: (i) cytotype 1 corresponds to three different taxa: *T. d. maculipennis* in Mexico (excluding Yucatán), *T. d. dimidiata* in Guatemala (excluding Petén) and probably also El Salvador; and *T. d. capitata* in Colombia; (ii) cytotype 2 was found in two localities (Paraiso and Chablekal) around Mérida, Yucatan, Mexico where the species *T*. sp. aff. *dimidiata* presents 5 different haplotypes (T.dim-H25, T.dim-H26, T.dim-H27, T.dim-H28 and T.dim-H31); (iii) cytotype 3 appeared in Yaxhá, Petén, Guatemala, where both *T. d. maculipennis* (T.dim-H18) and *T*. sp. aff. *dimidiata* (T.dim-H25, T.dim-H28 and T.dim-H30) are present. Sequencing of the same specimens studied [18] from Yaxhá showed that cytotype 3 was found in specimens of *T*. sp. aff. *dimidiata* of haplotype T.dim-H28 and T.dim-H30. Consequently, chromosomal cytotypes 2 and 3 are both found in *T*. sp. aff. *dimidiata*.

The two haplotypes of *T. hegneri* differ by only 3 mutations from haplotypes of *T. d. maculipennis*. This reduced number of nucleotide differences and the location of *T. hegneri* haplotypes within the clade of *T. dimidiata*, basal to haplotypes of group 2 (Figure 4), does not support its status as an independent species. The results obtained suggest that it is an insular form of *T. d. maculipennis*. Originally described from the island of Cozumel [3], a subspecific status *T. d. hegneri* could be maintained only if morphological characteristics allow a clear differentiation of the insular form, as the phylogenetic analysis somehow separates it in a very close but particular evolutionary line. *Triatoma hegneri*, although chromatically distinguishable from most forms of *T. dimidiata* [50], is known to produce fertile hybrids when experimentally crossed with *T. dimidiata* (R.E. Ryckman, unpublished). Interestingly, the most dispersed haplotypes of both *T. d. maculipennis* (T.dim-H18) and *T*. sp. aff. *dimidiata* (T.dim-H28) are also present on the same island, probably introduced through the intense human transport between the mainland and the island.

The distinction between *T. d. dimidiata* (subgroup 1A), *T. d. capitata* (subgroup 1B), *T. d. maculipennis* (group 2), *T*. sp. aff. *dimidiata* (group 3), and *T. d. hegneri* contributes giving systematic/taxonomic coherency to present knowledge about morphological and genetic concepts in these taxa. From an ancestral form close to *T*. sp. aff. *dimidiata*, it can be postulated that an original diversification focus of *T. dimidiata* forms took place most probably in Guatemala, with a southern spread into Panama and Colombia to give the *capitata* forms and a northwestern spread into Mexico to give the *maculipennis* forms (Figure 5). Thus, the results of the present paper, obtained from a large amount of samples of *T. dimidiata* from many different countries covering its whole latitude range, gives rise to a new frame that is different from the previous hypothesis about a clinal variation along a north-south axis, which was formerly suggested to explain both morphological data [50] and preliminary ITS-2 data from a reduced number of samples [6].

Moreover, the distinction between these five entities may facilitate the understanding of different vector transmission capacities and epidemiological characteristics of Chagas disease throughout the very large area where *T. dimidiata sensu lato* is distributed, from the Mexican northern latitude limit up to the Peruvian southern latitude limit [11]. Recent results obtained by means of a population dynamics model indicate that *T. dimidiata* in Yucatan, Mexico, is not able to sustain domestic populations, that up to 90% of the individuals found in houses are immigrants, and that consequently Chagas disease control strategies must be adapted to a transmission by non-domiciliated vectors [59]. This might be considered surprising because it does not fit the domiciliation capacity of *T. dimidiata* in other places, but it appears to be congruent if it is taken into account that in fact the Yucatan vector in question is not *T. dimidiata* but a different species *T*. sp. aff. *dimidiata*.

The results here obtained also suggest that *T. d. dimidiata* in Ecuador is a good candidate for the design of appropriate vector control intervention, similarly to domestic *T. infestans* populations in countries such as Uruguay, Chile and Brazil within the successful Southern Cone Initiative [60]. The control and even eradication of *T. d. dimidiata* in Ecuador by means of insecticide-spraying of its domestic habitats might be successful, if it is considered that it is merely an introduced vector species in that area, and a priori it would have difficulties in escaping from the insecticide activity because of its non-adaptativeness to the sylvatic environment in these two countries [61]. Unfortunately, such a control initiative will not be so easy to carry out in Colombia, as results prove that Colombian forms are authochthonous *T. d. capitata* and not *T. d. dimidiata* derived from the Ecuadorian introduced form. This fits with the existence of sylvatic populations in Colombia and with the high genetic similarity of sylvatic, peridomestic and domestic populations detected in that country [9]. Similarly to in Colombia, results indicate that *T. dimidiata* will offer, because of being authochthonous forms, more problems for insecticide-spraying control in Central American countries than introduced *T. infestans* in Southern Cone countries.

### The other Meso- and Central American *Triatoma* Species

*Triatoma bassolsae* differs by only one deletion from *T. pallidipennis* and appears in the branch of the 5 species traditionally included in the Phyllosoma complex: *T. longipennis*, *T. mazzotti, T. picturata*, *T. pallidipennis* and *T. phyllosoma*. The genetic differences between these taxa are so reduced (sometimes even none at all), that there is no support to maintain them as separated species. Such a low number of nucleotide differences in the ITS is considered as pertaining to organisms able to hybridize [62]. This fully fits the capacity of these taxa to crossbreed and give fertile hybrids [63],[64] and agrees with the entomologist conclusion of applying only subspecies level to them [49]. The divergence of members of the phyllosoma complex is estimated at only 0.74–2.28 Mya by the rDNA molecular clock [4], which also seems consistent with a subspecific rank. All further ITS-2 studies have always reached the same conclusion [5],[6],[65]. By analyzing many interfertility experiments [64], it can be concluded that, in triatomines, morphological differentiation appears to be faster than the installation of reproductive or genetic barriers [66],[67]. Rapid morphological changes, associated with ecological adaptation, helps to explain discordance between phenetic and genetic differentiation. Triatomine species with consistent morphological differences would arise through divergent ecological adaptation, a vision which fits with “evolutionary units” implying a different evolutionary direction taken by some populations [67]. Until future reproductive isolation thanks to ecological isolation is reached by these morphologically different entities of the Phyllosoma complex, the subspecies concept accurately fits for all these “evolutionary units” of the Phyllosoma complex. ITS-2 results indicate that *Triatoma bassolsae* is one additional taxon to be included in this situation, as has already been suggested [65]. The comparison of the small genetic divergences between these taxa, their distributions exclusively restricted to regions of Mexico, and their different geographical distribution areas slightly overlapping in their bordering zones [3] suggest that genetic exchange might be impeding or delaying definitive divergence processes to reach species level.

Genetic distances between the taxa of the Phyllosoma complex found when analyzing different mtDNA genes proved to be similar to those detected in ITS-2 at the 16S [68], but higher in CytB [65],[69], and COI [69]. This agrees with the evolutionary rates of the protein-coding mtDNA genes which are pronouncedly faster than the one of ITS-2. Moreover, aminoacid sequences of the CytB and COI genes show no one difference between the Phyllosoma complex members studied (all are silent mutations or synonymous substitutions) except one aminoacid difference between two populations of the same species *T. pallidipennis* and one in *T. picturata* versus the rest [69], which also fit with an intraspecific variability. Additionally, it shall be taken into account that (i) mtDNA becomes monophyletic more rapidly than does a single nuclear gene and far more rapidly than a sample of several nuclear genes, so that mtDNA may make inferences of species-level monophyly erroneous [70], and (ii) the known great potential of mtDNA to become monophyletic by selective sweeps can decrease the time to monophyly of a clade and not be reflective of the genealogical processes in the nuclear genome, advantageous mutations occurring on mtDNA causing the entire mitochondrial genome to become monophyletic because of the little or no recombination they have [71]. The crossbreeding capacity and hybrid viability among the Phyllosoma complex taxa in question is well known and, taking into account that their geographical distributions overlap in their border areas and there are no sufficient ecological differences indicating a local spatial separation, it becomes very difficult to support them as separate species from the evolutionary, biogeographical and ecological points of view because there is apparently no barrier for a reproductive isolation. Thus, the results of both ITS-2 and mtDNA genes fit with such an evolutionary, subspecific divergence, when taking into account the peculiarities of both nuclear and mitochondrial markers.

*Triatoma mexicana* appears to be a good species and its location in the phylogenetic tree fully supports its ascription to the Phyllosoma complex, similarly as suggested by a phylogentic analysis by means of a mtDNA CO1 fragment [69]. Surprisingly, *T. gerstaeckeri* (Rubrofasciata group) clusters with *T. mexicana*, suggesting that it should be included in the Phyllosoma complex. All these species, i.e. *T. phyllosoma* (including its subspecies *phyllosoma*, *longipennis*, *mazzotti, picturata*, *pallidipennis* and *bassolsae*), *T. dimidiata* (with its three subspecies *dimidiata*, *capitata* and *maculipennis*, to which *hegneri* shall be added), *T*. sp. aff. *dimidiata*, *T. mexicana* and *T. gerstaeckeri* constitute a well defined clade for which the generic taxon *Meccus*, proposed long ago [72], afterwards synonymized [50] and recently tentatively revalidated [73], seem to appropriately fit. Previous molecular studies, first with complete ITS-2 sequences [74] and second with partial mtDNA 16S gene sequences [68], also indicate that *Meccus* might be a valid taxon.

The revalidation of *Meccus*, as well as that of *Nesotriatoma* for species of the Flavida complex, has not been accepted because of the close relationship between *T. flavida* and the Phyllosoma complex [7]. The results of the present study do, however, pose a serious question concerning the inclusion of species as *T. bolivari* and *T. ryckmani* in the Phyllosoma complex, as they appear to cluster with *T. rubida* of the Rubrofasciata group with relatively high support (83 and 96 in ML and BPP, respectively). A *T. rubida* - *T. nitida* clade previously detected with weak support under certain conditions in mitochondrial DNA marker analyses [69] does not appear to be supported in the ITS-2 phylogeny.

Although not fully resolved in the tree obtained, the location of the Cuban *T. flavida* as a species basal to all other North-Central American *Triatoma* species may be interpreted as a consequence of being a relict insular species close to the ancient first North-Central American *Triatoma* colonizers. Further studies with other genetic markers are needed to establish the position of *T. flavida* more adequately.

### The South American *Triatoma* Species

The very scarce ITS-2 sequence differences between *T. arthurneivai* and *T. rubrovaria*, a species known in southern Brazil, Uruguay and northern Argentina [75], pose doubts on whether to keep the validity of *T. arthurneivai* as independent species. Recent genetic and morphometric studies have already raised several questions about *T. arthurneivai*, indicating that topotypes from Minas Geraes may represent a species different from populations of São Paulo State formerly also referred to *T. arthurneivai* and suggesting that these São Paulo populations might probably belong to *T. wygodzinskyi* [76]. This may explain the ITS-2 results, as the two specimens analyzed in the present paper come in fact from Espirito Santo do Pinhal, São Paulo State. Consequently, material of typical *T. wygodzinskyi* should be sequenced and compared to both true *T. arthurneivai* from Minas Geraes and *T. rubrovaria* to ascertain the status of these three taxa.

The South American *Triatoma* species cluster together with maximum support (100/100/100) and well separated from that of the North and Central American species of the same genus, thus supporting results of previous analyses which indicate an early divergence of about 23–38 Mya between species of the northern (Phyllosoma complex) and southern (*T. infestans*) continent [4],[6].

## Supporting Information

Alternative Language Abstract S1 Translation of the abstract into Spanish by S. Mas-Coma.
(0.03 MB DOC)

Table S1 Summary of analysis of molecular variance for *Triatoma dimidiata* populations.
(0.06 MB DOC)

Table S2 Summary of population genetic variation parameters from ITS-2 haplotypes in the *Triatoma dimidiata* populations.
(0.08 MB DOC)

Table S3 Evaluation of within groups genetic differentiation by computation of pairwise FST values for populations defined by country of origin in subgroup 1A.
(0.03 MB DOC)

Table S4 Summary of differentiation tests for *Triatoma dimidiata* populations based on ITS-2 haplotypes.
(0.06 MB DOC)
