# Supplementary material for: Phylogeography and Genetic Variation of Triatoma dimidiata, the Main Chagas Disease Vector in Central America, and Its Position within the Genus Triatoma
Source: PLoS Negl Trop Dis. 2008 May 7;2(5):e233. doi: 10.1371/journal.pntd.0000233 (PMC2330091; doi:10.1371/journal.pntd.0000233)
Supplement: Alternative Language Abstract S1 — Translation of the abstract into Spanish by S. Mas-Coma. (0.03 MB DOC) [file pntd.0000233.s001.doc]

**Resumen**

**Antecedentes.** La Enfermedad de Chagas, causada por *Trypanosoma cruzi*, sigue siendo una de las enfermedades parasitarias más serias de América Central y América del Sur. La contaminación humana en áreas pobres rurales y periurbanas se atribuye principalmente a insectos hemípteros hematófagos de la subfamilia Triatominae. *Triatoma* es el género más amplio, incluyendo especies de gran interés en salud pública debido a su importancia como vectores. *Triatoma dimidiata* es el vector principal en América Central y Ecuador. Esta especie se encuentra en habitats silvestres, peridomésticos y domésticos, actuando las poblaciones no domiciliadas como fuentes de reinfestación y viéndose así involucradas en la infestación humana. *Triatoma dimidiata* muestra una amplia diversidad fenotípica, genotípica y de comportamiento a lo largo y ancho de su distribución geográfica. En este amplio estudio se combinan análisis de secuencias de ADN, métodos de reconstrucción filogenética y estudios de variación genética para investigar los perfiles de haplotipos, polimorfismo genético, filogeografía y tendencias evolutivas de *T. dimidiata* desde México hasta Colombia y Ecuador, así como de las especies que le son más próximas dentro de *Triatoma*. Este trabajo representa el análisis interpoblacional más extenso jamás realizado sobre una especie de triatomino hasta la fecha.

**Metodología/Descubrimientos Pricipales.** Se utilizó un total de 165 triatominos procedentes de México, Guatemala, Honduras, Nicaragua, Panamá, Cuba, Colombia, Ecuador y Brasil, representando a 13 especies de *Triatoma*, dentro de las cuales muestras de *T. dimidiata* a partir de 64 orígenes geográficos diferentes. El espaciador ITS-2 del ADN ribosomal nuclear fue seleccionado como marcador genético por su conocida utilidad en Triatominae. La obtención de 137 secuencias del ITS-2 reveló la existencia de 31 haplotipos diferentes en *T. dimidiata*. Las poblaciones de *T. dimidiata* demuestran seguir divergencias evolutivas diferentes en las cuales el aislamiento geográfico parece haber tenido una influencia determinante. Una forma ancestral del Sur de México y Norte de Guatemala debió dar lugar a dos clados principales que incluyen a cuatro grupos/subgrupos. Una rama monofilética quedó confinada a la Península de Yucatán (incluyendo las islas de Cozumel y Holbox) y partes nórdicas del Estado de Chiapas, Guatemala y Honduras, con sus descendientes actuales mereciendo un rango de especie (*T*. sp. aff. *dimidiata*). Dentro de la segunda rama monofilética (desde México hasta Ecuador y Colombia), los patrones de diversidad poblacional, compatibles con una asignación de rango de subespecie, debieron conformarse probablemente por radiación adaptativa de poblaciones derivadas de formas ancestrales de Guatemala próximas a *T*. sp. aff. *dimidiata*. Las poblaciones de Centro América (Guatemala y Honduras y secundariamente México – costa del Pacífico en el Estado de Chiapas -, Nicaragua, Ecuador e isla de Providencia) se corresponden a la subespecie nominal *T. d. dimidiata*. Una expansión meridional hasta Panamá y Colombia dió lugar a la formas de *T. d. capitata* y otra expansión septentrional desde Guatemala hacia México a las formas de *T. d. maculipennis*. Se detectó varias introducciones presuntamente antropogénicas. *Triatoma hegneri* de la isla de Cozumel se muestra como una forma insular subespecífica de *T. dimidiata*. *Triatoma bassolsae* (sólo una forma de *T. phyllosoma*), *T. mexicana* y *T. gerstaeckeri* se agrupan filogenéticamente dentro del monofilético complejo Phyllosoma. Las muy escasas diferencias nucleotídicas detectadas sugieren la necesidad de revisar la validez de *T. arthurneivai* y *T. wygodzinskyi*.

**Conclusiones/Trascendencia:** La comparación con muy numerosas especies de *Triatoma*, tanto especies estrechamente relacionadas como filogenéticamente bien distantes, permite alcanzar conclusiones con alto soporte no únicamente sobre *T. dimidiata*, sino también sobre diferentes grupos importantes de especies de *Triatoma* y sus evoluciones. El análisis filogeográfico de las especies de *Triatoma* muestra dos lineas de colonización diferentes hacia el Norte y hacia el Sur del istmo de Panamá durante épocas pasadas. Una variabilidad genética intraespecífica tan amplia como la encontrada en la secuencia del ITS-2 de *T. dimidiata sensu lato* nunca había sido detectada en una especie de triatomino con anterioridad. La distinción entre los cinco taxones diferentes de *T. d. dimidiata*, *T. d. capitata*, *T. d. maculipennis*, *T. d. hegneri* y *T*. sp. aff. *dimidiata* aporta coherencia sistemático-taxonómica a los conocimientos actualmente disponibles sobre los conceptos morfológicos y genéticos en estos taxones y puede facilitar la comprensión de las diferentes capacidades de transmisión vectorial y diferentes características epidemiológicas de la Enfermedad de Chagas a lo largo y ancho de la amplísima área de su distribución en Centro y Meso-América. Esa distinción también indica que *T. dimidiata* va a presentar más problemas para el control de la enfermedad con rociados insecticidas de viviendas que en el caso de *T. infestans* en Uruguay, Chile y Brasil. Sin embargo, ello viene también a sugerir que las poblaciones de *T. dimidiata* en Ecuador (y probablemente también Perú) cabe considerarlas dianas apropiadas para fumigaciones insecticidas en respectivas estrategias de control.
