# Supplementary material for: Phylogeography and Genetic Variation of Triatoma dimidiata, the Main Chagas Disease Vector in Central America, and Its Position within the Genus Triatoma
Source: PLoS Negl Trop Dis. 2008 May 7;2(5):e233. doi: 10.1371/journal.pntd.0000233 (PMC2330091; doi:10.1371/journal.pntd.0000233)
Supplement: Table S1 — Summary of analysis of molecular variance for Triatoma dimidiata populations. (0.06 MB DOC) [file pntd.0000233.s002.doc]

**Table S1** Summary of analysis of molecular variance for *Triatoma dimidiata* populations

| Source of variation | d.f. | Sum of squares | Variance components | Percentage of variation | Fixation Indices |
| --- | --- | --- | --- | --- | --- |
| a) |  |  |  |  |  |
| Among groups | 2 | 528.273 | 6.781 Va | 80.87 | F_CT_ = 0.809*** |
| Among haplotypes within groups | 28 | 169.867 | 1.605 Vb | 19.13 |  |
| Within haplotypes | 105 | 0.0 | 0.0 Vc | 0.0 |  |
| Total | 135 | 698.140 | 8.386 |  |  |
| b) |  |  |  |  |  |
| Among groups | 1 | 68.257 | 1.3731 | 55.86 | F_CT_ =0.559*** |
| Among haplotypes within groups | 15 | 67.814 | 1.0852 | 44.14 |  |
| Within haplotypes | 68 | 0.0 | 0.0 | 0.0 |  |
| Total | 84 | 136.071 | 2.4580 |  |  |
| c) |  |  |  |  |  |
| Among groups | 3 | 596.530 | 5.704 | 84.15 | F_CT_ = 0.868*** |
| Among haplotypes within groups | 27 | 101.610 | 1.074 | 15.85 |  |
| Within haplotypes | 105 | 0.0 | 0.0 | 0.0 |  |
| Total | 135 | 698.140 | 6.783 |  |  |

(a) Three groups (1, 2, and 3), (b) two subgroups (1A vs 1B), and (c) four groups/subgroups (1A, 1B, 2 and 3) were considered as indicated in the text. ***: P < 0.001; **: P < 0.01. d.f.= degrees of freedom
