# Supplementary material for: Phylogeography and Genetic Variation of Triatoma dimidiata, the Main Chagas Disease Vector in Central America, and Its Position within the Genus Triatoma
Source: PLoS Negl Trop Dis. 2008 May 7;2(5):e233. doi: 10.1371/journal.pntd.0000233 (PMC2330091; doi:10.1371/journal.pntd.0000233)
Supplement: Table S2 — Summary of population genetic variation parameters from ITS-2 haplotypes in the Triatoma dimidiata populations. (0.08 MB DOC) [file pntd.0000233.s003.doc]

**Table S2**. Summary of population genetic variation parameters from ITS-2 haplotypes in the *Triatoma dimidiata* populations

| Parameter | Colomb1 | Colomb2 | Mex1 | Mex2 | Mex3 | Hond1 | Hond3 | Ecuador | Nicaragua | Guatem1 | Guatem2 | Guatem3 | Panama |
| --- | --- | --- | --- | --- | --- | --- | --- | --- | --- | --- | --- | --- | --- |
| Gene copies | 1 | 30 | 2 | 23 | 15 | 18 | 2 | 3 | 1 | 26 | 4 | 7 | 4 |
| Haplotypes | 1 | 5 | 2 | 7 | 5 | 4 | 1 | 2 | 1 | 6 | 1 | 3 | 2 |
| Polymorphic sites | 0 | 6 | 1 | 7 | 10 | 4 | 0 | 3 | 0 | 7 | 0 | 3 | 4 |
| Hap. diversity | NA | 0.602 | NA | 0.707 | 0.676 | 0.471 | NA | 0.667 | NA | 0.803 | NA | 0.524 | 0.500 |
| Std. error | NA | 0.071 | NA | 0.091 | 0.105 | 0.130 | NA | 0.314 | NA | 0.044 | NA | 0.209 | 0.265 |
| Pairw diff mean | NA | 1.248 | NA | 1.328 | 1.943 | 0.726 | NA | 2.000 | NA | 1.800 | NA | 0.857 | 2.000 |
| S.D. | NA | 0.812 | NA | 0.857 | 1.167 | 0.567 | NA | 1.512 | NA | 1.073 | NA | 0.682 | 1.405 |
| Nucleot diversity | NA | 0.003 | NA | 0.003 | 0.004 | 0.003 | NA | 0.004 | NA | 0.004 | NA | 0.002 | 0.004 |
| Std. error | NA | 0.002 | NA | 0.002 | 0.007 | 0.001 | NA | 0.004 | NA | 0.002 | NA | 0.002 | 0.003 |
| θ (Het) | NA | 1.139 | NA | 1.853 | 1.589 | 0.663 | NA | 1.519 | NA | 3.238 | NA | 0.822 | 0.747 |
| S.D. θ (Het) | NA | 0.347 | NA | 0.855 | 0.795 | 0.349 | NA | 2.237 | NA | 0.984 | NA | 0.697 | 0.801 |
| θ (k) | NA | 1.453 | NA | 3.042 | 2.203 | 1.278 | NA | 1.414 | NA | 2.132 | NA | 1.423 | 0.879 |
| 95 % C.I. for θ (k) | NA | 0.539,3.614 | NA | 1.245,7.094 | 0.771,5.976 | 0.419,3.609 | NA | 0.268,7.458 | NA | 0.839,5.083 | NA | 0.371,5.252 | 0.182,4.268 |
| θ (S) | NA | 0.505 | NA | 0.542 | 0.923 | 0.582 | NA | 1.333 | NA | 1.572 | NA | 0.408 | 1.091 |
| S.D. θ (S) | NA | 0.373 | NA | 0.403 | 0.598 | 0.435 | NA | 1.098 | NA | 0.785 | NA | 0.408 | 0.876 |
| θ (π) | NA | 1.248 | NA | 1.328 | 1.943 | 0.726 | NA | 2.000 | NA | 1.800 | NA | 0.857 | 2.000 |
| S.D. θ (π) | NA | 0.903 | NA | 0.955 | 1.308 | 0.634 | NA | 1.886 | NA | 1.195 | NA | 0.781 | 1.678 |
| Tajima’s D | NA | -1.256ns | NA | -0.408ns | -0.457ns | -0.740ns | NA | 0.0002ns | NA | -0.498ns | NA | -1.006ns | -0.710ns |
| Ewens-Watterson | NA | 0.418ns | NA | 0.323s | 0.369ns | 0.556ns | NA | 0.556ns | NA | 0.228ns | NA | 0.551ns | 0.625ns |
| Fu’s Fs | NA | -0.042ns | NA | -2.234ns | 0.126ns | -0.841ns | NA | 1.609ns | NA | -0.136ns | NA | -0.237ns | 2.197ns |

θ = effective mutation rate estimated from equilibrium heterozygosity [θ (Het)], number of alleles [θ (k)], number of polymorphic sites [θ (S)] and nucleotide diversity [θ (π)]. S.D. = standard deviation; C.I. = confidence interval
