# Supplementary material for: Phylogeography and Genetic Variation of Triatoma dimidiata, the Main Chagas Disease Vector in Central America, and Its Position within the Genus Triatoma
Source: PLoS Negl Trop Dis. 2008 May 7;2(5):e233. doi: 10.1371/journal.pntd.0000233 (PMC2330091; doi:10.1371/journal.pntd.0000233)
Supplement: Table S3 — Evaluation of within groups genetic differentiation by computation of pairwise FST values for populations defined by country of origin in subgroup 1A. (0.03 MB DOC) [file pntd.0000233.s004.doc]

**Table S3.** Evaluation of within groups genetic differentiation by computation of pairwise F_ST_ values for populations defined by country of origin in subgroup 1A

|  | Colombia1 | Mexico1 | Honduras1 | Ecuador | Nicaragua | Guatemala1 |
| --- | --- | --- | --- | --- | --- | --- |
| Colombia1 | - | - | - | - | - | - |
| Mexico1 | -1 | - | - | - | - | - |
| Honduras1 | 0.37815 | 0.53964* | - | - | - | - |
| Ecuador | -0.5 | 0.11765 | 0.59242*** | - | - | - |
| Nicaragua | 1 | 0.71429 | 0.82111 | 0.45455 | - | - |
| Guatemala1 | -0.61379 | -0.07942 | 0.19284*** | 0.24197* | 0.56262* | - |

Significance values (*: P < 0.05; **: P < 0.01; ***: P < 0.001) were derived by the exact differentiation test with a Markov chain of length 10000 steps
