# Supplementary material for: Phylogeography and Genetic Variation of Triatoma dimidiata, the Main Chagas Disease Vector in Central America, and Its Position within the Genus Triatoma
Source: PLoS Negl Trop Dis. 2008 May 7;2(5):e233. doi: 10.1371/journal.pntd.0000233 (PMC2330091; doi:10.1371/journal.pntd.0000233)
Supplement: Table S4 — Summary of differentiation tests for Triatoma dimidiata populations based on ITS-2 haplotypes. (0.06 MB DOC) [file pntd.0000233.s005.doc]

**Table S4.** Summary of differentiation tests for *Triatoma dimidiata* populations based on ITS-2 haplotypes

|  | Colombia1 | Colombia2 | Mexico1 | Mexico2 | Mexico3 | Honduras1 | Honduras3 | Ecuador | Nicaragua | Guatemala1 | Guatemala2 | Guatemala3 | Panama |
| --- | --- | --- | --- | --- | --- | --- | --- | --- | --- | --- | --- | --- | --- |
| Colombia1 | 0 | - | - | - | - | - | - | - | - | - | - | - | - |
| Colombia2 | 0.74174*** | 0 | - | - | - | - | - | - | - | - | - | - | - |
| Mexico1 | -1 | 0.76992*** | 0 | - | - | - | - | - | - | - | - | - | - |
| Mexico2 | 0.87326 | 0.86247*** | 0.06747 | 0 | - | - | - | - | - | - | - | - | - |
| Mexico3 | 0.90864* | 0.92555*** | 0.91438* | 0.94169*** | 0 | - | - | - | - | - | - | - | - |
| Honduras1 | 0.37815 | 0.75375*** | 0.53964* | 0.8937*** | 0.9381* | 0 | - | - | - | - | - | - | - |
| Honduras3 | 1 | 0.94285*** | 0.97778 | 0.95468*** | 0.2822 | 0.96819** | 0 | - | - | - | - | - | - |
| Ecuador | -0.5 | 0.78226*** | 0.11765 | 0.87379*** | 0.91367* | 0.59242*** | 0.94313 | 0 | - | - | - | - | - |
| Nicaragua | 1 | 0.84065*** | 0.71429 | 0.85988 | 0.91994 | 0.82111 | 1 | 0.45455 | 0 | - | - | - | - |
| Guatemala2 | 1 | 0.87615*** | 0.97598* | -0.06132 | 0.94009* | 0.9348*** | 1 | 0.92429* | 1 | 0.08987 | - | - | - |
| Guatemala1 | -0.61379 | 0.68882*** | -0.07942 | 0.85146*** | 0.91202* | 0.19284*** | 0.92179*** | 0.24197* | 0.56262* | 0 | 0.84764*** | - | - |
| Guatemala3 | 0.95946 | 0.94087*** | 0.95941* | 0.95447*** | -0.03385 | 0.96295*** | 0.52897* | 0.94878*** | 0.9645 | 0.92346*** | 0.97822*** | 0 | - |
| Panama | 0.66667 | 0.42124 | 0.7346 | 0.86474*** | 0.90753* | 0.8193*** | 0.93239 | 0.72727* | 0.77778 | 0.6955*** | 0.9* | 0.9409*** | 0 |

The lower hemimatrix corresponds to pairwise FST values for the corresponding populations and values in the diagonal correspond to within population diversity. Significance values (*: P < 0.05; **: P < 0.01; ***: P < 0.001) were derived by the exact differentiation test with a Markov chain of length 10000 steps
